# The *Salmonella* Kinase SteC Targets the MAP Kinase MEK to Regulate the Host Actin Cytoskeleton

**DOI:** 10.1016/j.chom.2012.09.011

**Published:** 2012-11-15

**Authors:** Charlotte Odendall, Nathalie Rolhion, Andreas Förster, John Poh, Douglas J. Lamont, Mei Liu, Paul S. Freemont, Andrew D. Catling, David W. Holden

**Affiliations:** 1 Section of Microbiology, MRC Centre for Molecular Bacteriology and Infection, Imperial College London, London SW7 2AZ, UK; 2 Centre for Structural Biology, Division of Molecular Biosciences, Imperial College London, London SW7 2AZ, UK; 3 FingerPrints Proteomics Facility, College of Life Sciences, University of Dundee, Dundee DD1 5EH, Scotland, UK; 4 Department of Pharmacology and Stanley S. Scott Cancer Center, Louisiana State University Health Sciences Centre, New Orleans, LA 70112, USA

## Abstract

After host cell entry, *Salmonella* replicate in membrane-bound compartments, which accumulate a dense meshwork of F-actin through the kinase activity of the *Salmonella* SPI-2 type III secretion effector SteC. We find that SteC promotes actin cytoskeleton reorganization by activating a signaling pathway involving the MAP kinases MEK and ERK, myosin light chain kinase (MLCK) and Myosin IIB. Specifically, SteC phosphorylates MEK directly on serine 200 (S200), a previously unstudied phosphorylation site. S200 phosphorylation is predicted to displace a negative regulatory helix causing autophosphorylation on the known MEK activatory residues, S218 and S222. In support of this, substitution of S200 with alanine prevented phosphorylation on S218 and S222, and phosphomimetic mutations of S200 stimulated phosphorylation of these residues. Both *steC*-null and kinase-deficient mutant strains displayed enhanced replication in infected cells, suggesting that SteC manipulates the actin cytoskeleton to restrain bacterial growth, thereby regulating virulence.

## Graphical abstract

**Graphical Abstract:**

**Figure:**
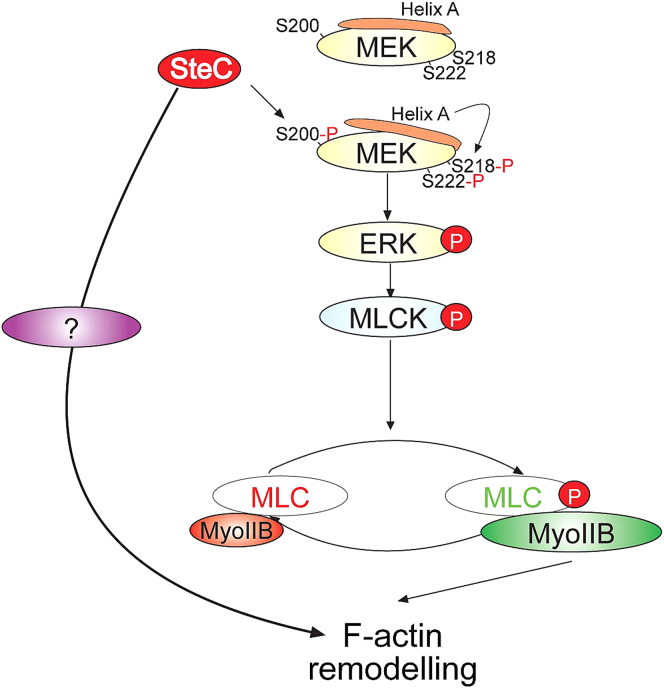

**Highlights:**

► *Salmonella* SteC reorganizes F-actin via a MEK/ERK/MLCK/Myosin IIB pathway ► SteC phosphorylates MEK1 S200, which is required for SteC-induced MEK activation ► MEK1 S200 phosphorylation induces autophosphorylation on residues S218/222 ► SteC also controls bacterial growth via its kinase function

## Introduction

*Salmonella enterica* serovar Typhimurium (*S*. Typhimurium) causes gastrointestinal disease in humans and a typhoid-like systemic infection in certain mouse strains. Its virulence requires two type III secretion systems (T3SSs), encoded by the pathogenicity islands *Salmonella* Pathogenicity Island 1 (SPI-1) and SPI-2. T3SSs are multiprotein organelles assembled in the bacterial cell envelope with needle-like extensions (Cornelis and Van Gijsegem, 2000). Effectors proteins translocated by the SPI-1 T3SS interfere with the actin cytoskeleton to induce bacterial invasion and contribute to the early maturation of the *Salmonella*-containing vacuole (SCV) (Steele-Mortimer et al., 2002). The SCV matures into an organelle that has some properties of late endosomes but lacks degradative enzymes of lysosomes (Garcia-del Portillo and Finlay, 1995). A few hours after bacterial entry, the SPI-2 T3SS is activated and delivers a second set of effector proteins across the vacuolar membrane. These further modify the SCV and enable intravacuolar bacterial replication (Haraga et al., 2008; Helaine et al., 2010). In the course of these events, *S*. Typhimurium causes major alterations to the microtubule, intermediate filament (Guignot et al., 2004), and actin (Méresse et al., 2001; Miao et al., 2003) networks. Through the action of the SPI-2 T3SS effector SteC, it induces the formation of a meshwork of F-actin around SCVs and bacterial microcolonies (Poh et al., 2008). Although this protein was shown to have kinase activity (Poh et al., 2008), its target(s) and the physiological significance of the actin meshwork it forms remained unknown. A previous study showed that small GTPases of the Rho family, N-WASP, Scar/WAVE, the Arp2/3 complex, and mDia1, are not involved in SPI-2-mediated actin reorganization (Unsworth et al., 2004). Overexpression of SteC in 3T3 fibroblasts induced the formation of thick F-actin bundles connected to clusters of highly condensed F-actin (Poh et al., 2008). This stellate appearance is very similar to that obtained after expression of a constitutively active mutant of rho-activated kinase (ROCK) (Amano et al., 1996; Poh et al., 2008), which causes Myosin II activation through myosin light chain (MLC) phosphorylation (Gallagher et al., 1991). This suggests that SteC-dependent actin reorganization might involve the formation of F-actin cables through the action of Myosin II. Here we show that SteC activates a pathway containing MEK, ERK, myosin light chain kinase (MLCK), and Myosin IIB. We show that SteC activates MEK1 via a phosphorylation mechanism different to that of endogenous MEK kinases. Deletion of *steC* or its kinase activity led to a modest increase in intracellular replication of *S*. Typhimurium in epithelial cells and macrophages and bacterial growth in mice. We conclude that phosphorylation of MEK by SteC contributes to the formation of the SCV-associated actin meshwork and that this could serve to restrain bacterial growth and regulate bacterial virulence.

## Results

### Phosphorylated Myosin IIB Is Recruited to SteC-Dependent F-Actin Structures

**Figure 1:**
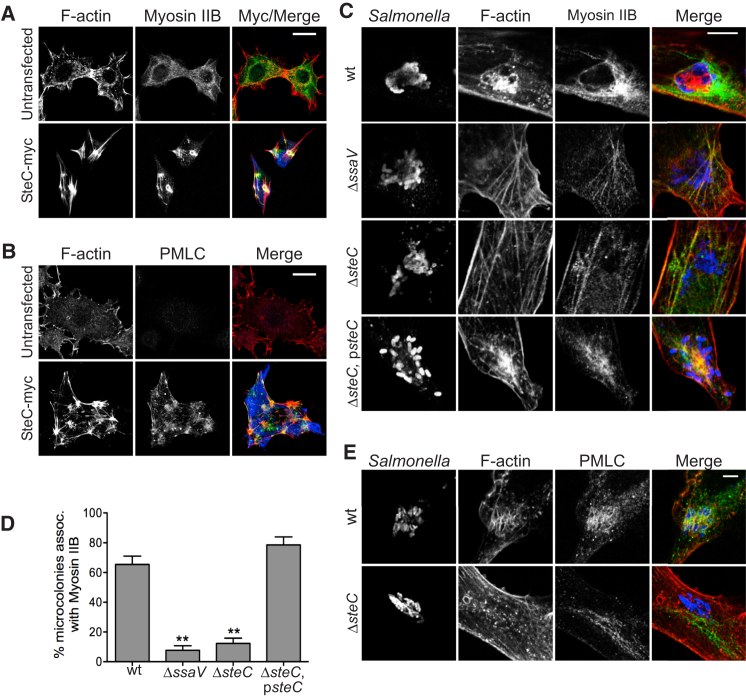
Myosin II Is Recruited to Sites of SteC-Dependent F-Actin Reorganization in Its Phosphorylated, Activated Form (A and B) Swiss 3T3 fibroblasts expressing SteC-myc (blue) were labeled for Myosin IIB (green in A) or phospho-myosin light chain (PMLC, green in B) and stained for F-actin (red). Scale bars represent 40 μm (A) or 20 μm (B). (C–E) Swiss 3T3 fibroblasts were infected with different strains of *S*. Typhimurium (blue) as indicated and were labeled for Myosin IIB (green in C) or PMLC (green in E) and F-actin (red). Scale bars represent 8 μm. The percentage of microcolonies associated with Myosin IIB was quantified in (D). Results are expressed as means ± SEM of four independent experiments. ^∗∗^p < 0.01. See also Figure S1.

SteC overexpression causes the formation of thick F-actin fibers connected to clusters of highly condensed F-actin, similar in appearance to those obtained when Myosin II is activated (Poh et al., 2008). Three isoforms of the heavy chain of Myosin II are expressed in nonmuscle cells. Little is known about Myosin IIC (Buxton et al., 2003; Golomb et al., 2004), but Myosin IIA and Myosin IIB are well characterized. Myosin IIA was not detected on SCVs in HeLa cells (Wasylnka et al., 2008). Since Myosin IIB localizes to a more central region of nonmotile fibroblasts (Saitoh et al., 1987), we examined the distribution of the heavy chain of Myosin IIB in Swiss 3T3 cells expressing SteC. In all cells examined, SteC expression caused the formation of very thick F-actin bundles to which Myosin IIB was recruited (Figure 1A). Myosin II molecules are hexamers composed of heavy-chain dimers and two pairs of light chains. Phosphorylation of the regulatory MLC induces activation of the protein complex, and activated Myosin II can bind and bundle F-actin filaments (Bresnick, 1999). Accordingly, Swiss 3T3 cells were labeled with an antibody recognizing phosphorylated MLC (PMLC). Untransfected, serum-starved cells displayed little to no cytosolic PMLC labeling (Figure 1B). In cells expressing SteC, PMLC labeling was enhanced throughout the cell and always colocalized with F-actin fibers and clusters (Figure 1B). Therefore, SteC is sufficient to promote phosphorylation of MLC and its recruitment to SteC-dependent F-actin bundles.

Swiss 3T3 cells were also infected with different strains of *S*. Typhimurium and examined for recruitment of the Myosin IIB heavy chain to SCVs. In cells infected for 8 hr with wild-type (WT) or complemented deletion mutant (Δ*steC*, p*steC*) strains, approximately 70% of microcolonies were associated with Myosin IIB (Figures 1C and 1D). In cells infected with *ssaV* (SPI-2 T3SS defective) or *steC* mutant bacteria, Myosin IIB association with SCVs was much lower. The *steC* mutant translocated other effectors as efficiently as WT *Salmonella* (data not shown). This shows that SteC is responsible for the recruitment of Myosin IIB to bacterial microcolonies. PMLC colocalized with F-actin structures around WT *S*. Typhimurium vacuoles (Figure 1E), but no PMLC was found in the vicinity of vacuoles containing *steC* mutant bacteria, indicating that SteC is also essential for the recruitment of PMLC to SCVs.

Pharmacological inhibitors were used to investigate whether Myosin II is involved in SteC-dependent F-actin accumulation. To avoid prolonged exposure of cells and bacteria to the inhibitors (which could result in nonspecific effects and loss of inhibitor activity), we pretreated infected cells with the actin-depolymerizing agent Latrunculin B, which completely prevents SCV-associated F-actin structures (Unsworth et al., 2004). After Latrunculin B washout, F-actin accumulates in the vicinity of vacuoles containing WT but not *ssaV* (Unsworth et al., 2004) or *steC* mutant (data not shown) bacteria. Therefore the Myosin inhibitors BDM or Blebbistatin were added during Latrunculin B washout. Exposure of cells to either drug decreased their ability to accumulate F-actin in the vicinity of SCVs (Figure S1 available online), indicating that Myosin II is involved in the process of SteC-induced F-actin bundling.

### Myosin Isoform Specificity in SteC-Induced F-Actin Reorganization

**Figure 2:**
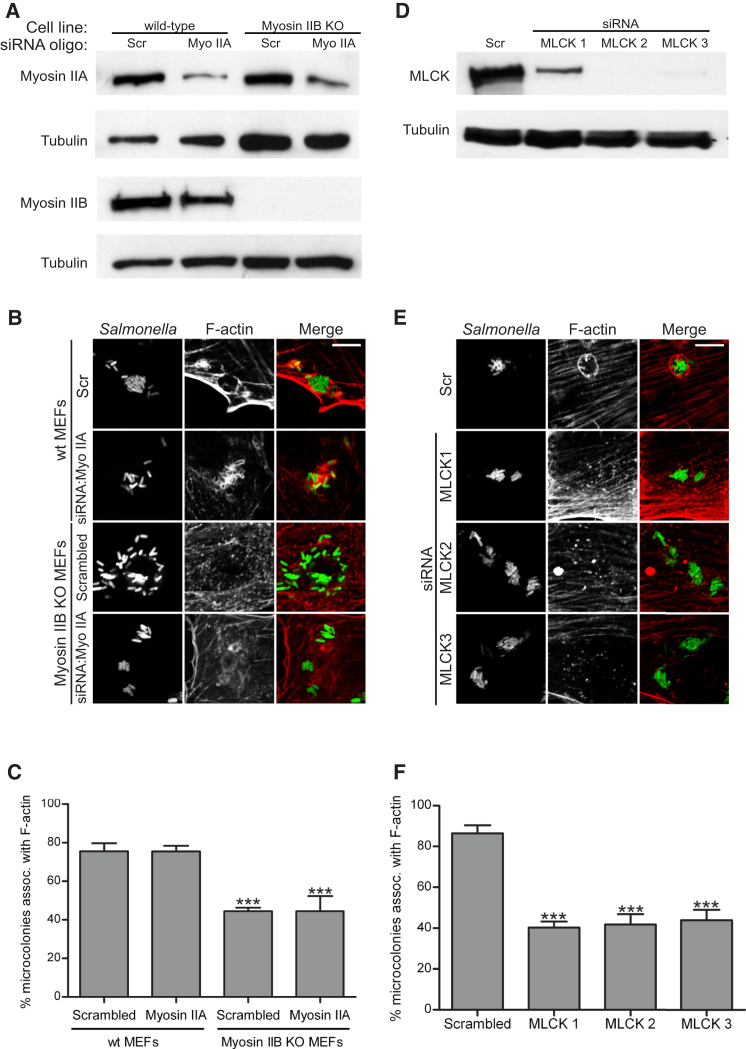
Myosin IIB and MLCK Are Involved in SteC-Dependent F-Actin Remodeling RNAi-mediated knockdown of Myosin IIA in WT or Myosin IIB KO cells (A–C) or of myosin light chain kinase (MLCK) (D–F). (A) WT or Myosin IIB KO mouse embryonic fibroblasts (MEFs) were transfected with scrambled (Scr) or Myosin IIA (Myo IIA)-specific pools of siRNA oligos, as indicated. Myosin IIA and Myosin IIB levels were assessed by WB. The same blots were probed for tubulin as a loading control. (D) Swiss 3T3 fibroblasts were transfected with a Scr oligo or three individual siRNA oligos against MLCK (denoted MLCK1, MLCK2, and MLCK3). Whole-cell lysates were analyzed by WB against MLCK or tubulin. (B and E) siRNA-treated cells were infected with GFP-expressing WT *S*. Typhimurium (green) and stained for F-actin (red). Scale bars represent 8 μM. (C and F) Percentage of *Salmonella* microcolonies associated with F-actin in the conditions indicated. Results are expressed as means ± SEM of a least three independent experiments. ^∗∗∗^p < 0.001. See also Figure S2.

To study the requirement of Myosin II isoforms in the formation of SteC-dependent F-actin meshworks, we used mouse embryonic fibroblasts (MEFs) lacking Myosin IIB. These cells and their WT counterparts do not express Myosin IIC (Lo et al., 2004). Control cells and Myosin IIB knockout (KO) cells were depleted of Myosin IIA by RNA interference (RNAi)-mediated knockdown (Figure 2A) and infected with WT bacteria for 8 hr. Only cells that were clearly depleted of Myosin IIA were analyzed. In WT MEFs transfected with a scrambled small interfering RNA (siRNA) oligo, 75.5% ± 4% of microcolonies were associated with F-actin. Myosin IIA knockdown in WT cells had no effect on SteC-dependent F-actin accumulation: 75.4% ± 3% of microcolonies were associated with F-actin in these cells (Figures 2B and 2C). However, in Myosin IIB KO MEFs, only 44.4% ± 2% of microcolonies were associated with F-actin (Figure 2C), showing that this isoform contributes to SteC-dependent F-actin meshwork formation. Since the inhibition of SteC-dependent F-actin bundling was not complete, we carried out RNAi of Myosin IIA in these cells, to investigate the possibility of functional redundancy between the two isoforms. However, there was no additive effect of Myosin IIA depletion in Myosin IIB KO cells (Figure 2C). Additionally, embryonic stem cells lacking Myosin IIA (Even-Ram et al., 2007) displayed similar levels of F-actin association as control cells (data not shown), showing that Myosin IIB, and not Myosin IIA, is involved in SteC-dependent F-actin accumulation. The incomplete reduction in F-actin association with SCVs in the absence of Myosin II suggests that SteC also activates a Myosin-independent pathway to promote F-actin reorganization.

### MLCK, ERK, and MEK Contribute to SteC-Dependent F-Actin Structures

MLC phosphorylation and subsequent Myosin II activation is regulated by numerous kinases, the best described of which are MLCK and ROCK (Amano et al., 1996; Kamm and Stull, 1985). We used the inhibitors ML-7 and Y27632 in the context of Latrunculin B washouts to investigate whether MLCK and/or ROCK are involved in SteC-dependent F-actin accumulation. ROCK inhibition had no effect on SteC-dependent F-actin accumulation (Figure S2A). However, exposure of cells to ML-7 led to a reduction in SCV-associated F-actin from 73.2% ± 7% in control cells to 40% ± 1% in treated cells, indicating that MLCK is likely to be involved in this pathway.

To clarify the roles of MLCK and ROCK in SteC-dependent F-actin reorganization, we carried out RNAi-mediated knockdown of these proteins in Swiss 3T3 fibroblasts (Figure S2B; Figure 2D). Single or combined knockdown of ROCK I and ROCK II isoforms had no detectable effect on SteC-dependent F-actin reorganization (Figure S2C). However, depletion of MLCK with three individual oligos reduced F-actin accumulation around microcolonies by approximately 50% (Figures 2E and 2F; Figure S2C), confirming a role for MLCK in this process.

MLCK is activated upon Ca^2+^/calmodulin binding, and this activation was shown to be enhanced by ERK-mediated phosphorylation (Klemke et al., 1997). As the amino acid sequence of SteC displays similarity to that of C-Raf (Poh et al., 2008), a member of the Raf/MEK/ERK MAP kinase pathway (Seger and Krebs, 1995), the roles of MEK and ERK in SteC-dependent F-actin reorganization were investigated.

**Figure 3:**
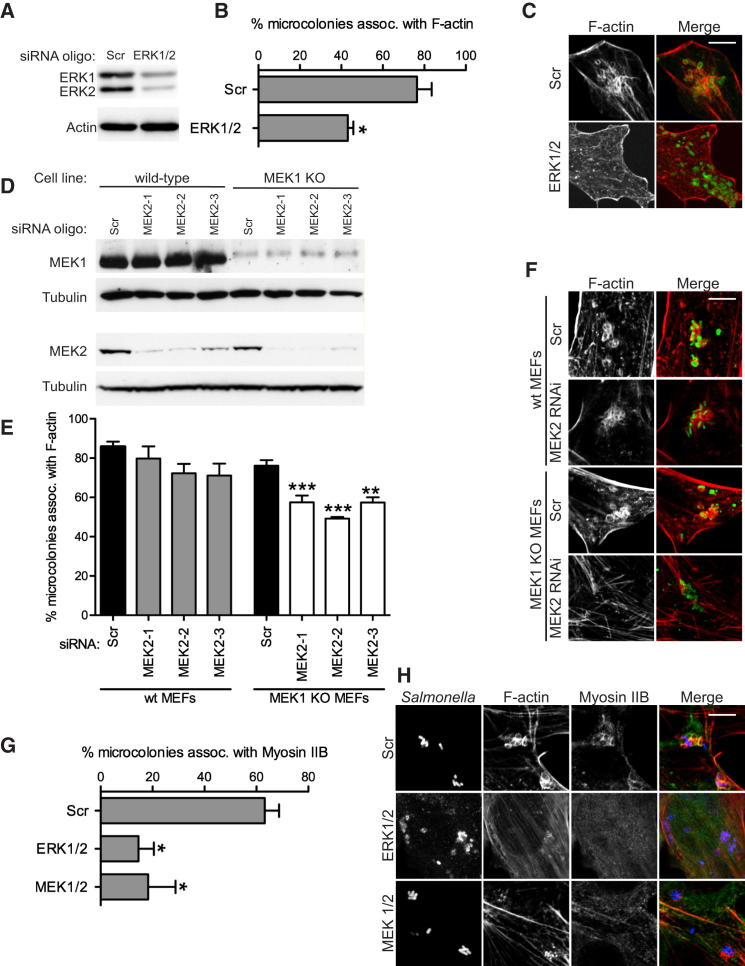
MEK and ERK Contribute to F-Actin Meshwork Formation (A–C) WT MEFs were depleted of ERK1 and ERK2 with a mixture of siRNA oligos. Cells were infected with GFP-expressing *S*. Typhimurium (green in C), stained for F-actin (red) and scored for F-actin association with microcolonies (B). (D–F) Similarly, WT or MEK-1 KO MEFs were transfected with a scrambled (Scr) oligo or three individual MEK2-targeting siRNAs (denoted MEK2-1, MEK2-2, and MEK2-3) as indicated (D). Cells were infected with *Salmonella* (green in F) and stained for F-actin (red), and F-actin association with microcolonies was quantified (E). (G and H) MEFs depleted of ERK1/2 or MEK1/2 were infected for 8 hr with WT *S*. Typhimurium (blue in H) and were labeled for Myosin IIB (green) and stained for F-actin (red). Myosin IIB recruitment to *Salmonella* microcolonies was quantified (G). Results are expressed as means ± SEM of three independent experiments (B, E, and G). Scale bars represent 8 μm (C, F, and H). ^∗^p < 0.05, ^∗∗^p < 0.01, ^∗∗∗^p < 0.001. See also Figure S3.

Swiss 3T3 cells were infected with WT *S.* Typhimurium and subjected to Latrunculin B washouts in the presence of MEK1/2 (PD98059) or ERK (FR180204) inhibitors. Both inhibitors significantly reduced the percentage of microcolonies associated with F-actin (Figures S3A and S3B). The involvement of these proteins was also investigated with siRNA. Efficient depletion of ERK1 and ERK2 (Figure 3A) led to a decrease in the percentage of SCV-associated F-actin from 76.4% ± 7% to 43.2% ± 3% (Figures 3B and 3C). To deplete cells of both MEK isoforms, we subjected MEK2 to siRNA in WT and MEK1 KO fibroblasts (Figure 3D). RNAi-mediated knockdown of MEK2 in WT cells caused a very limited reduction in SCV-associated F-actin compared to cells transfected with a scrambled siRNA oligo (Figures 3E and 3F), and microcolonies remained associated with F-actin in MEK1 KO cells. However RNAi of MEK2 in MEK1 KO fibroblasts led to a significant decrease in SCV association with F-actin (Figures 3E and 3F). Together, these results show that MEK and ERK are involved in SteC-dependent F-actin assembly and that MEK1 and MEK2 are functionally redundant in this pathway.

The experiments above indicate that MEK, ERK, MLCK, and Myosin II each contribute partially to SCV-associated F-actin. To study whether MEK, ERK, and Myosin are in the same SteC-induced pathway, we examined SCV-associated Myosin IIB in cells depleted of ERK1/2 or MEK1/2. There was a strong reduction of Myosin IIB recruitment to microcolonies in cells lacking these proteins (Figures 3G and 3H). In addition, exposure of fibroblasts expressing SteC to inhibitors of MEK or MLCK prevented phosphorylation of Myosin and completely blocked the formation of SteC-induced F-actin fibers. However, some SteC-dependent F-actin-rich structures remained (indicated by arrowheads in Figure S3C). Evidently, SteC-induced F-actin meshwork formation involves one pathway requiring MEK, ERK, MLCK, and Myosin II and a second pathway that is independent of these proteins.

Next we investigated whether a Raf isoform might be involved in SteC-induced F-actin structures. RNAi of B-Raf and C-Raf was carried out in Swiss 3T3 fibroblasts (Figure S4A), which were then infected with WT *S.* Typhimurium. Although both isoforms could be effectively depleted singly or in combination (Figure S4A), a similar proportion of microcolonies was associated with F-actin in knocked down and control cells, indicating that neither isoform is involved in this process (Figures S4B and S4C).

### SteC Promotes MEK and ERK Phosphorylation

**Figure 4:**
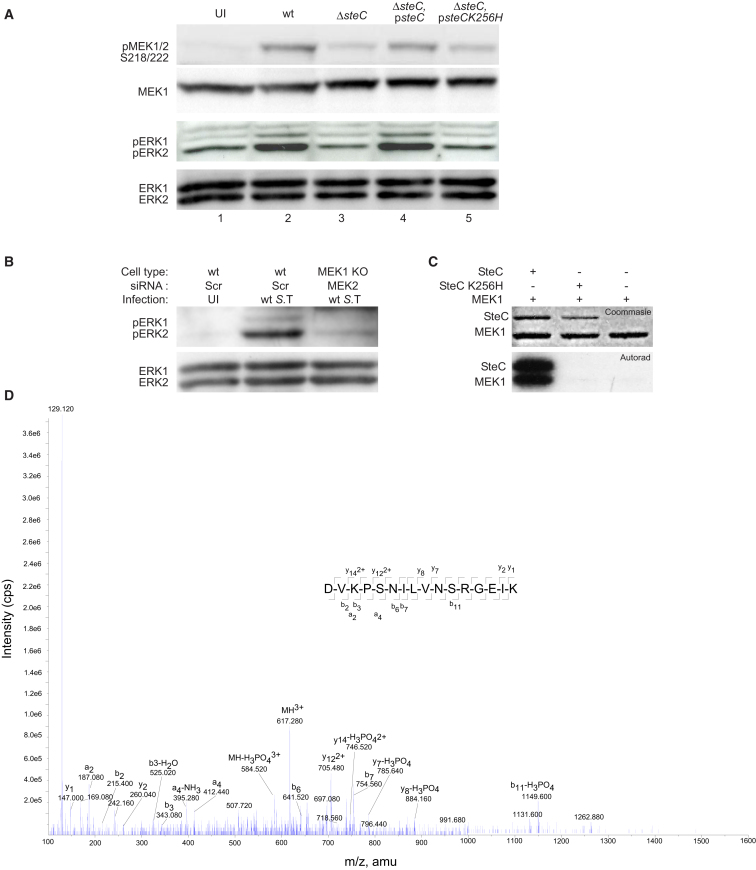
Phosphorylation of MEK1 by SteC (A) Swiss 3T3 fibroblasts were infected with the indicated strains of *S*. Typhimurium (*S*. T) for 8 hr. Cells were then lysed and MEK1/2 or ERK1/2 phosphorylation was assessed by WB with antibodies specific for MEK1/2 phosphorylated on S218/S222 or ERK phosphorylated at the MEK (T202/T204) consensus site. Total MEK or ERK levels were assessed as loading controls. (B) WT or MEK1 KO MEFs were transfected with scrambled (Scr) or MEK2-targeting siRNA oligos. Cells were then infected with WT *S*. Typhimurium for 8 hr and ERK phosphorylation was measured as described above. (C) MEK1 was incubated with SteC or SteCK256H in kinase assay buffer with [γ^-32^P] ATP at 30°C for 30 min. Proteins were subjected to SDS-PAGE followed by Coomassie blue staining and autoradiography. (D) Phosphorylation site identification of MEK phosphopeptide DVKPSNILVNSRGEIK. Tandem mass spectrum obtained for precursor ion at m/z 617.280^3+^ was used in conjunction with MS-Product (ProteinProspector v 5.7.2) to annotate both C-terminal (y) ions and N-terminal (b) ions present and confirm the site of phosphorylation (denoted by S). The y1 ion indicates that the peptide has a Lys (K) residue at its C terminus and the b2/b3 ions correlate with the N terminus of the peptide, Asp-Val-Lys (D-V-K). The loss of phosphoric acid (MH-H3PO4^3^+) from the triply charged precursor (MH^3+^) indicates that the peptide is phosphorylated and the site of phosphorylation is confirmed by the ions b6/b7 and y7-H3PO4 and y8-H3PO4. m/z, mass to charge ratio in atomic mass units (amu); cps, counts per second. See also Figure S4.

To examine SteC-dependent phosphorylation of MEK during bacterial infection, we infected Swiss 3T3 fibroblasts with different strains of *S.* Typhimurium for 8 hr and subjected lysates to immunoblotting with an antibody recognizing MEK1 and MEK2 phosphorylated on the two activatory residues: S218 and S222. Infection with WT bacteria greatly increased the levels of phosphorylated MEK, in an SteC-dependent manner (Figure 4A, top panel, compare lanes 2 and 3). Expression of SteC in the *steC* mutant strain restored MEK phosphorylation. This was kinase dependent since infection with the Δ*steC*, p*steCK256H* (kinase dead) (Poh et al., 2008) strain did not restore the ability of the *steC* mutant strain to phosphorylate MEK (Figure 4A, top panel, lanes 4 and 5). ERK phosphorylation was tested with an antibody recognizing both ERK1 and ERK2 phosphorylated on the MEK target sequence. As for MEK, ERK was phosphorylated by infection with WT bacteria in an SteC- and SteCK256-dependent manner (Figure 4A, lower panels). Furthermore, cells depleted of both MEK1 and MEK2 had much reduced *Salmonella*-induced ERK phosphorylation (Figure 4B). Taken together, these results show that SteC induces the phosphorylation of MEK and subsequent ERK phosphorylation in infected cells.

The similarity between the kinase domain of SteC and C-Raf (Poh et al., 2008) suggested that SteC might mimic C-Raf function and phosphorylate MEK directly. To test this, we purified SteC and the kinase-dead mutant SteCK256H as 6-Histidine (His) fusion proteins. These were incubated with [γ^-32^P] ATP and purified WT MEK1. Proteins were then separated by SDS-PAGE and analyzed by autoradiography. SteC underwent autophosphorylation as described previously (Poh et al., 2008). Whereas SteCK256H was unable to autophosphorylate or phosphorylate MEK1 (Figure 4C), ^32^P was incorporated into MEK1 following incubation with SteC, showing that MEK1 is directly phosphorylated by SteC in vitro (Figure 4C).

Phosphorylation of MEK1 by SteC was then analyzed by mass spectrometry with precursor ion scanning. Phosphorylation of either S218 or S222 was revealed by the presence of a weakly assigned phosphopeptide. However, a combination of precursor ion scanning and titanium dioxide enrichment clearly revealed phosphorylation of S200 (Figure 4D). This residue is conserved in both MEK1 and MEK2 of mammalian species, as well as in Ste7 in *Saccharomyces cerevisiae* (data not shown).

### SteC Induces MEK1 Autophosphorylation

**Figure 5:**
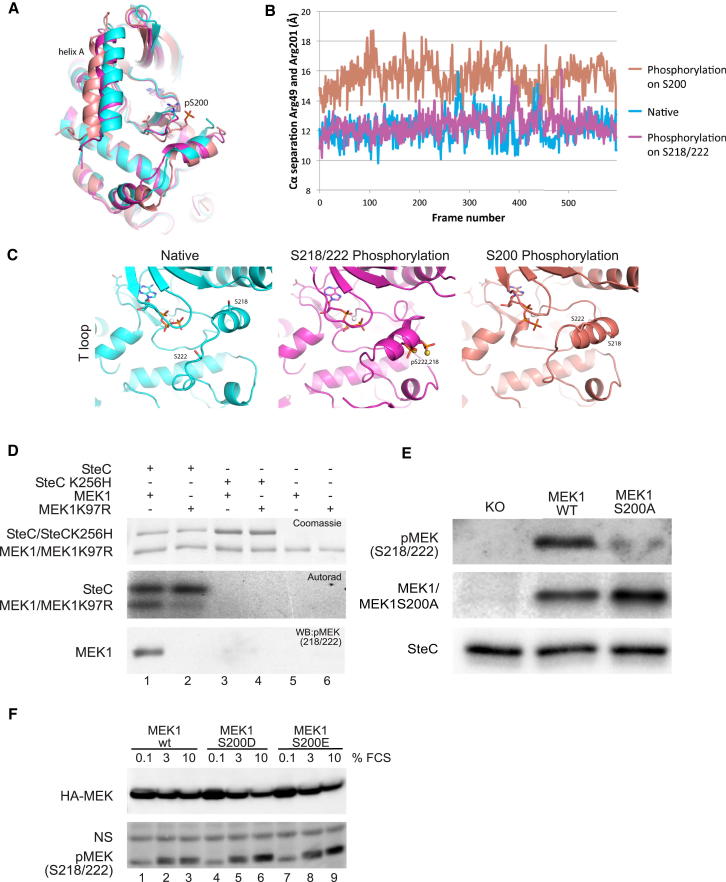
MEK1 Phosphorylation on S200 Induces Helix A Displacement and Autophosphorylation on S218/222 (A–C) Molecular dynamics (MD) simulations were carried out on native MEK1 (cyan), MEK1 phosphorylated on S218/222 (magenta), or MEK1 phosphorylated on S200 (salmon). All snapshots were taken at 35.6 ns. (A) Superposition of three snapshots showing helix A displacement when S200 is phosphorylated. Helix A was excluded for the calculation of the superposition. (B) Separation between the alpha carbon atoms of R49 and R201 over the course of the simulation. (C) Cartoon representation of the region around the T loop. S218, S222 and ATP are shown as sticks, Mg^2+^ as a white sphere. In the middle panel, two stabilizing Na^+^ ions are shown as yellow spheres. (D) MEK1 or MEK1K97R were incubated with SteC or SteCK256H in kinase assay buffer with [γ^-32^P] ATP at 30°C for 30 min. Proteins were subjected to SDS-PAGE followed by Coomassie blue staining and autoradiography or WB with a phospho-specific antibody against MEK1 phosphorylated on S218 and S222. (E) MEK1 variants were immunoprecipitated from MEK1 KO cells expressing MEK1-Flag (MEK1-WT) or MEK1 S200A-Flag. Cells containing no transgene (KO) were used as a control. IP mixtures were incubated with recombinant SteC-His in kinase assay buffer at 30°C for 30 min. MEK1 phosphorylation on S218/222 was assessed by WB. The membrane was reprobed with anti-Flag and anti-His antibodies as loading controls. (F) REF52 cells expressing HA-MEK1, HA-MEK1 S200D, or HA-MEK1 S200E were serum starved for 16 hr. The indicated concentrations of fetal calf serum (FCS) were added to the growth medium for 10 min. MEK proteins were immunoprecipitated with an anti-HA antibody and assessed for their phosphorylation on S218/222 by WB. Membranes were reprobed with an HA antibody, as a loading control. See also to Figure S5.

Structural analysis of MEK revealed that amino acids 32–51 fold into an alpha helix (helix A) that is packed against the N-terminal lobe and the MEK1 kinase domain and maintains MEK1 in an inactive state (Fischmann et al., 2009; Mansour et al., 1996; Ohren et al., 2004). S200 is part of a loop that forms critical interactions with the helix: R49 on helix A interacts with R201 (Figure S5), the interaction being further stabilized by E203 (Fischmann et al., 2009). Mutations of residues 47–49 or 203 increase MEK1 activity (Delaney et al., 2002; Mansour et al., 1996). Therefore, the interaction between these residues is important for the packing of helix A against the MEK1 kinase domain, and disruption of these interactions induces MEK1 activation. To investigate whether addition of a negative phosphate group on S200 by SteC might alter these interactions, we carried out molecular dynamics analyses using the unmodified protein (native), MEK1 phosphorylated in silico at position S200, or on the consensus regulatory residues S218/222. While the whole-protein geometry remained largely unchanged over time, important changes occurred in two specific regions of the protein, helix A and the T loop. The stacking interaction between R49 and R201 was broken in MEK1 phosphorylated on S200 (Figure S5). This caused a displacement of helix A (Figure 5A) measured by an increase from 12.9 Å to 16.9 Å between the alpha carbons of R49 and R201 (Figure 5B; Figure S5).

In native MEK1, the T loop carrying the two activatory residues, S218 and S222, had a poorly defined conformation and appeared flexible (Figure 5C). When both S218 and S222 were phosphorylated, the T loop peptide backbone assumed a helical conformation in which the serine residues were largely fixed in space. Strikingly, S200 phosphorylation appeared to induce a similar, rigid T loop structure with S218 and S222 in a short helix. Therefore, S200A partially mimicked the conformational change brought onto the T loop upon direct phosphorylation of S218/222.

Helix A displacement was previously shown to induce MEK1 activation, in part by inducing autophosphorylation on S218/222 (Mansour et al., 1996). Since S200 phosphorylation is predicted to induce a conformational change in the T loop, we hypothesized that this might favor autophosphorylation on S218/222. To test this, the ability of SteC to cause phosphorylation of MEK1 on S218/222 was examined in an in vitro kinase assay. Total phosphorylation of MEK1 by SteC was assessed by autoradiography after incubation of the proteins in the presence of radiolabeled ATP. As described above, SteC but not kinase-dead SteCK256H induced MEK1 phosphorylation (Figure 5D, middle panel). Use of a phosphospecific antibody for S218/222 showed that SteC induced MEK1 phosphorylation on S218/222 (Figure 5D, bottom panel, lane 1). To test whether this could occur indirectly through autophosphorylation, we incubated SteC with kinase-inactive MEK1 (MEK1K97R, Figure 5D, lane 2). Total phosphorylation of MEK1K97R by SteC was observed, but levels were repeatedly very low when compared to phosphorylation of WT MEK1 (Figure 5D, middle panel, lanes 1 and 2). However, phosphorylation of MEK1K97R on S218/222 was never observed (Figure 5D, bottom panel, lane 2). These results suggest that SteC requires MEK1 kinase activity to induce phosphorylation on S218/222 and that SteC might activate MEK1 by inducing its autophosphorylation on the T loop serines.

To determine whether S200 is necessary for SteC-mediated MEK1 autophosphorylation, we used retroviral gene transfer of MEK1 KO MEFs to create cell lines expressing similar levels of WT MEK1 or a point mutant of MEK1 in which S200 was substituted with alanine (S200A). WT MEK1 and MEK1 S200A were immunoprecipitated and subjected to in vitro kinase assays in the presence of recombinant His-SteC. Whereas SteC caused phosphorylation of S218/222 of WT MEK, it was unable to induce efficient phosphorylation of S218/222 in MEK1S200A (Figure 5E) (p < 0.05). This shows that S200 is important for SteC-mediated MEK1 autophosphorylation on its activatory residues.

To further characterize S200-mediated MEK1 autophosphorylation, we created MEK1 phosphomimetic mutants by substituting S200 with aspartic acid (S200D) or glutamic acid (S200E). These constructs were expressed in serum-starved REF52 fibroblasts (Park et al., 2007). Cells were then incubated in 0.1%, 3%, or 10% FCS for 10 min (Figure 5F). Ectopically expressed MEK1 proteins were immunoprecipitated and S218/222 phosphorylation was assessed by immunoblotting. WT MEK1 displayed modest S218/222 phosphorylation upon serum stimulation. By contrast, the S200D or S200E mutants displayed stronger S218/222 phosphorylation in presence of 3% or 10% serum. Therefore, mimicking S200 phosphorylation by SteC potentiates conventional phosphorylation of MEK1 on S218/222. However, since the phosphomimetic mutants did not stimulate S218/222 phosphorylation in the absence of serum, we conclude that phosphorylation of S200 is necessary but not sufficient for SteC-induced activation of MEK.

### SteC Controls Intracellular Replication of *S.* Typhimurium

**Figure 6:**
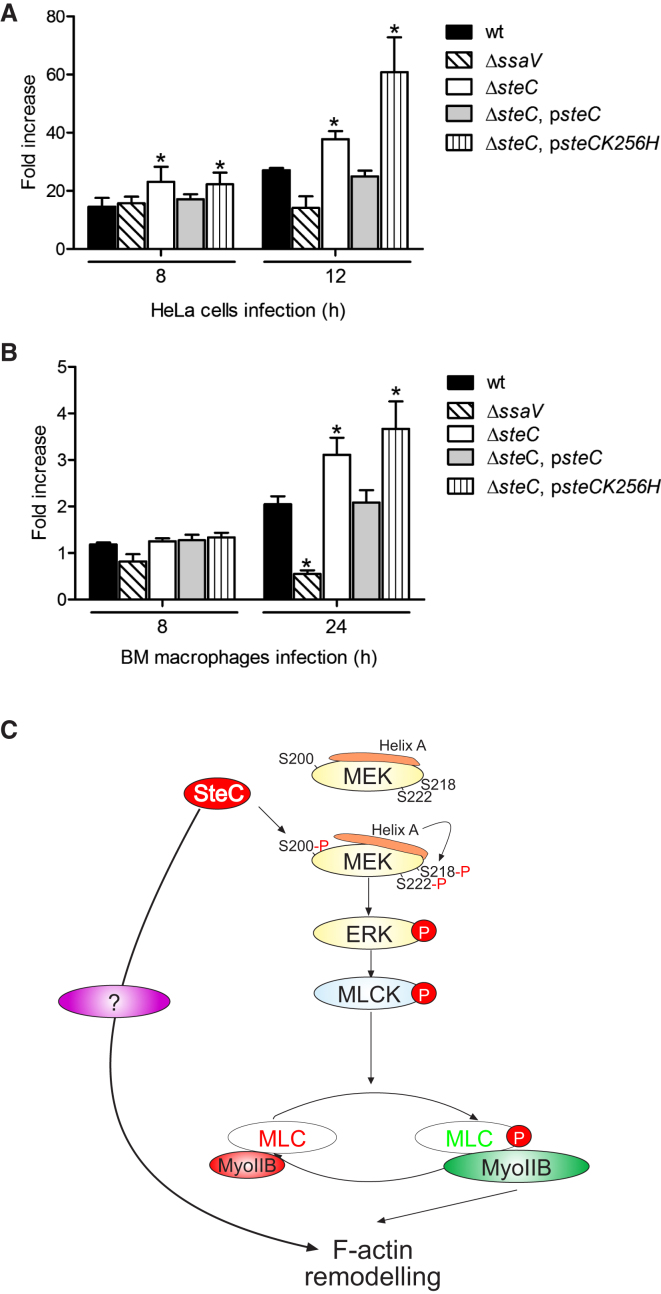
SteC Controls *Salmonella* Replication (A and B) HeLa cells (A) or bone marrow (BM)-derived macrophages (B) were infected with the indicated *S*. Typhimurium strains. At the time points indicated, cells were lysed, and released bacteria were grown on rich medium for enumeration of colony-forming units (cfu). Values of fold increase in bacterial numbers were calculated from the ratio of the numbers of cfu/ml at each time point, and the number of intracellular bacteria at 2 hr after entry. Results are expressed as mean fold increase ± SEM of at least three independent experiments. ^∗^p < 0.05. (C) Model of SteC-dependent F-actin reorganization: SteC activates MEK through phosphorylation of S200, causing displacement of helix A and autophosphorylation on S218/222. MEK1 phosphorylation leads to the activation of a signal transduction pathway involving ERK, MLCK, and Myosin IIB to induce cytoskeletal rearrangements in the vicinity of *Salmonella* microcolonies. SteC also induces a Myosin-independent signaling pathway to remodel the actin cytoskeleton.

Previous work failed to reveal a virulence defect of the *steC* mutant strain in the mouse model of systemic infection (Poh et al., 2008). To reassess the possibility of an effect of SteC on virulence, we inoculated mice by the oral route with a mixed inoculum comprising equivalent numbers of WT and *steC* mutant bacteria and compared their growth after 4 days by competitive index (CI) analysis, which provides a sensitive measure of the relative degree of attenuation (Beuzón and Holden, 2001). The *steC* mutant had a mean CI of 4.33 ± 1.1, showing that its growth in vivo was greater than that of the WT strain. This is consistent with results obtained elsewhere (Geddes et al., 2007). As *S.* Typhimurium replicates poorly in fibroblasts (Cano et al., 2001), we measured its growth in both epithelial cells and macrophages. After 12 hr in HeLa cells, there were approximately twice as many WT as *ssaV* mutant bacteria (Figure 6A). Similar results were obtained in mouse bone marrow (BM)-derived macrophages (Figure 6B). Interestingly, there was a significantly greater number of *steC* mutant bacteria compared to WT and Δ*steC,* p*steC* bacteria after both 8 hr and 12 hr growth in HeLa cells and after 24 hr in BM macrophages (Figures 6A and 6B). The Δ*steC*, p*steCK256H* mutant also displayed increased intracellular growth, showing that this effect was dependent on the kinase activity of SteC.

## Discussion

To date, approximately 28 effectors translocated by the *Salmonella* SPI-2 T3SS have been identified (Figueira and Holden, 2012), but the biochemical activities and physiological functions of most remain to be established. In this paper, we describe a signaling pathway activated by the SPI-2 T3SS effector SteC to promote the formation of host cell F-actin structures that accumulate close to SCVs in infected cells. Using a variety of different approaches, we show that SteC activates a pathway involving MEK, ERK, MLCK, and Myosin IIB (Figure 6C). We found that SteC activates MEK in a noncanonical manner by phosphorylating a previously unstudied residue, which is predicted to cause the displacement of an inhibitory helix and autophosphorylation of MEK1 on its activatory residues. In addition, we have shown that SteC acts to restrain bacterial growth in infected cells and in mice.

Whereas the Raf/MEK/ERK signaling pathway is well studied in relation to cell proliferation, apoptosis, differentiation, and immune responses (Shaul and Seger, 2007), its involvement in cytoskeletal dynamics is less well understood, even though there is increasing evidence that MEK/ERK regulate focal adhesion dynamics and promote cell migration (Anand-Apte et al., 1997; Brahmbhatt and Klemke, 2003; Fincham et al., 2000; Klemke et al., 1997; Lai et al., 2001; Nguyen et al., 1999; Webb et al., 2001). In particular, ERK was shown to phosphorylate MLCK directly, increasing its capacity to phosphorylate MLC and activate Myosin II to promote cell migration (Klemke et al., 1997; Morrison et al., 1996). The work presented here provides additional evidence for a role of MEK and ERK in cytoskeletal dynamics, and for the MEK/ERK/MLCK/Myosin II pathway.

This pathway has not been shown previously to be targeted by bacterial pathogens. However, several other virulence factors—including *Salmonella* SPI-1 and SPI-2 T3SS effectors—manipulate individual components of the MEK/ERK/MLCK/Myosin II pathway. For example, the SPI-1 effector SopB was shown to target Myosin II to facilitate both *Salmonella* entry into cells (Hänisch et al., 2011) and positioning in the perinuclear region (Wasylnka et al., 2008). However, in both cases SopB activates Myosin II via a pathway that involves the small GTPase Rho and its effector ROCK. Although SteC also modulates Myosin II activity, it is highly unlikely to be involved in the above processes. First, translocation of SteC was first detected 4 hr after *S*. Typhimurium entry (Poh et al., 2008), and second, loss of SteC does not affect the localization of SCVs in host cells (data not shown). In addition, SopB, together with the SPI-1 effectors SopE and SopE2, induces the phosphorylation of ERK, causing intestinal inflammation (Bruno et al., 2009). ERK is also negatively regulated by the SPI-2 T3SS effector SpvC (Mazurkiewicz et al., 2008), a phosphothreonine lyase that was shown to dephosphorylate ERK and other MAP kinases. Interestingly, SpvC was only detected in infected cells 16 hr after entry. This is of interest as SCV-associated F-actin structures are no longer detectable after 18 hr of infection (Brumell et al., 2002). This has been attributed to SpvB (encoded within the *spv* operon immediately upstream of *spvC*), which ADP ribosylates actin and inhibits the formation of SCV-associated actin structures (Miao et al., 2003), but it is possible that SpvC also contributes to this process by reversing SteC-dependent ERK phosphorylation.

MEK1 is known to autophosphorylate or be phosphorylated by Raf, MEKK, and Mos on two serines found on the T loop: S218 and S222 (Alessi et al., 1994). This is predicted to cause the rotation of the N-proximal lobe and the C-proximal lobes with respect to each other, closing the catalytic cleft. This is accompanied by stabilization of interactions between the activatory loop and active site residues, optimizing the orientation of residues required for ATP binding and catalysis of the γ phosphate of ATP onto the target protein (Cox et al., 1994; Goldsmith and Cobb, 1994; Johnson et al., 1996). Our evidence suggests that unlike Raf, SteC activates MEK1 indirectly, by inducing its autophosphorylation on S218/222. This is reminiscent of PAK1-mediated activation of MEK1: PAK1 was shown to phosphorylate MEK1 on S298, causing its autophosphorylation on S218/222 (Park et al., 2007). However, SteC phosphorylates MEK on S200. This residue is part of a loop that contains R201 and E203, which interact with R49 on helix A (Fischmann et al., 2009). This is of particular interest as helix A was previously shown to inhibit MEK1 by packing onto the kinase domain (Mansour et al., 1996). In agreement with this, deletions of residues within the helix, or substitution of E203 stimulated MEK1 activity by several orders of magnitude (Delaney et al., 2002; Mansour et al., 1996). Molecular dynamics analyses revealed that phosphorylation of MEK1 on S200 caused helix A to move away from the rest of protein, which is predicted to derepress MEK1 activity. It also induced a conformational change to the T loop carrying S218/222, even though this loop is situated 30 Å away from S200. We propose that S200 phosphorylation leads to S218/222 autophosphorylation by inducing two conformational changes: the displacement of helix A and the repositioning of the T loop. Although substitution of S200 with an alanine residue prevented efficient activation of MEK by SteC, phosphomimetic substitutions only stimulated phosphorylation on S218/222 in the presence of serum after starvation. Therefore, phosphorylation of S200 is necessary but unlikely to be sufficient for SteC-induced activation of MEK. Furthermore, inhibition and absence of some of the proteins in the MEK/ERK/MLCK/Myosin II pathway only reduced SCV-associated F-actin by approximately 50%. This suggests the existence of an actin reorganization activity of SteC that is independent of the Myosin pathway reported here. It is possible that other effectors exist that are dependent on SteC and modulate the actin cytoskeleton independently of the Myosin pathway.

It is becoming clear that while the majority of *Salmonella* virulence proteins that interfere with host cell functions promote bacterial growth, others appear to restrain its proliferation (Baek et al., 2009; Gal-Mor et al., 2008; Ho and Slauch, 2001). SteC seems to belong to the second category. The rate of intracellular bacterial growth appears to be a tightly controlled process involving a balance between the activity of replication-promoting and replication-restraining virulence proteins.

How the kinase activity of SteC functions to modulate intracellular growth of *Salmonella* is unknown, but several possibilities can be envisaged. First, it has been shown that in macrophages, *S.* Typhimurium activates MEK to restrain bacterial growth (Rosenberger and Finlay, 2002), while other studies have reported that it induces ERK-dependent IL-10 production (Uchiya et al., 2004; Uchiya and Nikai, 2004) and inhibits proinflammatory cytokine production (Uchiya and Nikai, 2005). It is possible that the kinase activity of SteC contributes to these phenomena. Second, it is likely that the SteC-driven recruitment of Myosin II and F-actin to SCVs interferes with normal vesicular traffic and fusion events in the host cell. Myosin II is required for vesicle biogenesis at the Golgi (Ikonen et al., 1997; Miserey-Lenkei et al., 2010), and actin and myosins are involved in short-range movement and fusion of vesicles with late endocytic organelles (Kjeken et al., 2004). Since SCVs interact with both endocytic (Méresse et al., 1999) and Golgi-derived (Mota et al., 2009) vesicles, the reorganization of Myosin II and F-actin could influence SCV maturation and bacterial growth. Third, SteC-mediated activation of MEK and ERK is likely to have consequences beyond cytokine production and actin reorganization, since these proteins affect many cellular processes. Further work is required to clarify the function of this interesting protein in restraining growth of *S.* Typhimurium in host cells.

## Experimental Procedures

### Bacterial Strains, Plasmids, and Growth Conditions

The bacterial strains used in this study are listed in Table S1. Bacteria were grown in Luria Bertani medium (LB) at 37°C in an orbital shaker. Where appropriate, LB was supplemented with 50 μg/ml carbencillin (Cb) or 50 μg/ml kanamycin (Km). The plasmids used in this study are listed in Table S2. Plasmids were purified with a QIAfilter plasmid midi or mini kit (QIAGEN). Site-directed mutagenesis was done as described in the Supplemental Experimental Procedures.

### Antibodies

A list of primary antibodies used in this study is shown in Table S3. AMCA-, Cy2-, Cy5-, or Rhodamine red X (RRX)-conjugated donkey anti-goat, anti-rabbit, or anti-mouse antibodies (Jackson Immunoresearch Laboratories) were used for immunofluorescence (IF) at a dilution of 1:200. RRX-conjugated phalloidin was purchased from Molecular Probes and used at a dilution of 1:100. Anti-mouse, anti-goat, anti-rabbit, and anti-rat (IgG) horseradish peroxidase-conjugated secondary antibodies (Amersham Pharmacia Biosciences) were used at a dilution of 1:10,000 for western immunoblotting (WB) analysis.

### Cell Culture, Transfection, and Retroviral Gene Transfer

Please refer to the Supplemental Experimental Procedures.

### Bacterial Infection of Cells, Immunofluorescence Microscopy, and Replication Assays

HeLa cells, MEFs, and Swiss 3T3 fibroblasts were infected with exponential phase *S*. Typhimurium as described previously (Beuzón et al., 2000). For infection of BM macrophages, bacteria were grown in LB overnight. After opsonization in mouse serum for 20 min, bacteria were added to the cells at a multiplicity of infection of ∼10:1, centrifuged at 110 × g for 5 min, and incubated at 37°C for 25 min. So that a synchronized population of bacteria could be followed, extracellular bacteria were killed through washing of the host cells with DMEM containing 100 μg/ml gentamicin for 1 hr. The concentration of gentamicin was then decreased to 20 μg/ml for the remainder of the experiment. For enumeration of intracellular bacteria, infected cells were washed three times with PBS and lysed with 0.1% Triton X-100 for 5 min, and dilution series were plated onto LB agar. All inhibitors were purchased from Calbiochem. In experiments involving Latrunculin B washouts, 4 hr after invasion, infected cells were incubated Latrunculin B (1 μg/ml) for 4 hr. They were then washed thoroughly with fresh medium and incubated with ML-7, Y27632, PD98059 (30 μM), FR180204 (20 μM), BDM (5 mM), or Blebbistatin (25 μM) for 30 min. For IF, cells were fixed in paraformaldehyde, permeabilized in 0.1% saponin, and incubated with antibodies as described (Beuzón et al., 2000). Labeled cells were analyzed with a Zeiss confocal laser scanning microscope (LSM510) or a Nikon T-2000 inverted spinning disc confocal microscope (Yokogawa).

### Protein Purification and Kinase Assays

SteC-6His and SteCK256H-6His were expressed and purified as described before (Poh et al., 2008). Protein samples were dialysed in 40 mM Tris-Cl (pH 7.4) overnight and used for kinase assays. For assays with recombinant MEK proteins, 1 μg purified SteC-6His or SteCK256H-6His was incubated with 1 μg MEK1 or MEK1K97R (Upstate). Assays were carried out in 50 mM Tris-Cl (pH 7.5), 10 mM MgCl_2_, 100 mM NaCl, and 1 mM dithiothreitol. In radioactive assays, 20 μM ATP and 2 μCi [γ^−32^P] ATP (Amersham, 370 MBq ml^−1^, 3000 Ci mmol^−1^) were added, or 1 mM ATP was used in cold assays. The mixture was incubated for 30 min at 30°C and subjected to SDS-PAGE, Coomassie blue staining, and autoradiography or WB with phospho-specific antibodies. Alternatively, MEK1 KO cells transduced with vectors encoding MEK1-Flag or MEK1-S200A-Flag were lysed in modified RIPA buffer (50 mM Tris-Cl, 250 mM NaCl, 0.25% Sodium deoxycholate, 1% NP-40, 1 mM Na_3_VO_4_, 1 mM NaF, and Complete EDTA-containing protease inhibitor). MEK1 variants were immunoprecipitated with agarose-conjugated mouse anti-Flag antibody (M2, Sigma). Kinase assays were carried out as described above, directly on the beads.

### Cell Lysis and Western Blotting

Swiss 3T3 cells or MEFs were collected from a well in a 6-well plate 3 days after transfection with siRNAs. Infected Swiss 3T3 cells were collected 8 hr after invasion. Cells were lifted with trypsin and washed in PBS before lysis in 5× sample buffer (0.25 M Tris-Cl [pH 6.8], 10% SDS, 10% β-Mercaptoethanol, 10% glycerol, and 0.05% Bromophenol Blue) supplemented with 2 mM activated Na_3_VO_4_ and 10 mM NaF. Samples were boiled for 5 min and separated by SDS-PAGE. Samples were then transferred onto PVDF Immobilon-P membranes (Millipore), and WB was carried out according to the manufacturer’s instructions. Animal procedures were conducted in accordance with UK Home Office Guidelines.

### Competitive Index Assay

Female BALB/c mice (B and K Universal, Hull, UK) of 18–22 g were inoculated by oral gavage with bacteria suspended in physiological saline solution. The bacterial inocula used were 1 × 10^8^ cfu of each strain in 0.2 ml. At least five mice were inoculated per strain mixture for each experiment. Mice were sacrificed 4 days after inoculation. The CI value is the mean of three independent experiments.

### Mass Spectrometry and Molecular Dynamics Analysis

Please refer to the Supplemental Experimental Procedures.

### Statistical Analyses

All results are reported as mean ± standard error of the mean (SEM). Statistical analyses were performed with Prism 5 software (GraphPad) with one-way ANOVA and Bonferroni post hoc analyses or two-tailed unpaired Student’s t test. Differences denoted as significant in the text fall below a p value of 0.05.

## Supplemental Information

Document S1. Supplemental Experimental Procedures, Figures S1–S5, and Tabled S1–S3
