# Supplementary material for: The Salmonella Kinase SteC Targets the MAP Kinase MEK to Regulate the Host Actin Cytoskeleton
Source: Cell Host Microbe. 2012 Nov 15;12(5):657–68. doi: 10.1016/j.chom.2012.09.011 (PMC3510437; doi:10.1016/j.chom.2012.09.011)
Supplement: Document S1. Supplemental Experimental Procedures, Figures S1–S5, and Tabled S1–S3 [file mmc1.pdf]

Cell Host & Microbe, Volume *12*

Supplemental Information

The *Salmonella* Kinase SteC Targets the MAP Kinase MEK to Regulate the Host Actin Cytoskeleton

Charlotte Odendall, Nathalie Rolhion, Andreas Förster, John Poh, Douglas J. Lamont, Mei Liu, Paul S. Freemont, Andrew D. Catling, and David W. Holden

Supplemental Experimental Procedures

Site-directed mutagenesis

Site-directed mutagenesis was performed by inverse PCR using pCMV-HA-MEK1 as DNA template. Ser at residue 200 of MEK1 was changed to Asp using 2FMEK1S200D (CCAACATTCTAGTGAACGATCGTGGGGAGATCAAACTC) and 2RMEK1S200D (CAGAGTTTGATCTCCCCACGATCGTTCACTAGAATGTTGG), to Glu using 2FMEK1200E (CCAACATTCTAGTGAACGAACGTGGGGAGATCAAACTC) and 2RMEK1S200E (CAGAGTTTGATCTCCCCACGTTCGTTCACTAGAATGTTGG) or to Alanine using 2FMEK1S200A (CCAACATTCTAGTGAACGCCCGTGGGG AGATCAAACTC) and 2RMEK1S200A (GAGTTTGATCTCCCCACGGGCGTT CACTAGAATGTTGG).

Cell culture, transfection and retroviral gene transfer

HeLa (clone HtA1) cells were obtained from the European Collection of Cell Cultures, Salisbury, UK. Swiss 3T3 murine fibroblast cells were kindly provided by Emmanuelle Caron (Imperial College London, UK). MEFs KO for MEK1 and their associated wt controls were a gift from Manuela Baccarini (University of Vienna, Austria). MEFs KO for Myosin IIB and their parental lines were provided by Robert Adelstein (National Heart, Lung, and Blood Institute, Maryland, USA). Cells were cultured in DMEM according to standard techniques. Primary bone marrow-derived macrophages were obtained from BALB/c mice (Racoosin and Swanson, 1989). Cells were grown in complete medium: RPMI (GIBCO, Carlsbad, CA) supplemented with 10% FCS (Foetal Calf Serum), 2 mM glutamine, 1 mM Na pyruvate, 10 mM HEPES, 50 µM β-mercaptoethanol, 100 U/ml penicillin/streptomycin and 20% L929-cells conditioned medium (LCM) (National Institute for Medical Research, Mill Hill, UK).
Plasmids were transfected into Swiss 3T3 cells using jetPEI (Polyplus Transfection), following the manufacturer’s recommendations. After 20 h, cells were transferred into serum-free DMEM for 4 h, fixed and labelled as described below.
Expression and analysis of MEK constructs in REF52 fibroblasts was performed as previously described (Park et al., 2007). After 24 h, cells were transferred into DMEM containing 0.1% FCS for 16 h before stimulation with FCS (final concentration 3 or 10%) for 10 min.
MEK alleles were introduced into MEK1 KO MEFs by retroviral gene transfer and then sorted for equal GFP fluorescence to normalize to MEK expression levels.
With the exception of siRNAs targeting B- and C-Raf which were obtained from Qiagen, all siRNA oligos were purchased from Ambion (Applied Biosystems).

Transfection of siRNA oligos into Swiss 3T3 fibroblasts and MEFs was carried out using Lipofectamine RNAiMAX (Invitrogen), following the manufacturer's instructions. Briefly 6 µM RNAiMAX was used to transfect 10 nM siRNA oligo for 48 h.

Mass spectrometry

Kinase assays were carried out as described above, with 10 µg of MEK1. After SDS-PAGE and Coomassie staining (Bio-Safe, BioRad), the band containing MEK1 was excised, processed, in-gel digested with trypsin and subsequently analysed by precursor ion scanning on a 4000 QTRAP as described previously (Sullivan et al., 2008). Enrichment of phosphopeptides from the MEK1 protein digest by titanium dioxide was carried out using as previously described (Thingholm et al., 2006). The titanium enriched phosphopeptides were then analysed on an Ultimate U3000 nLC system (Dionex) coupled to a LTQ Orbitrap XL (Thermo Scientific) using a proxeon ion source. The phosphopeptides were separated using a 65 min linear gradient from 5-40% acetonitrile in 0.1% formic acid. A Top5 methodology (FT-MS at 60,000 resolution with 5 IT-MS-MS scans) was used using a mass range between 335 and 1800 m/z. The data was extracted using Raw2MSM (ver1.7_2007.04.11) before database searching with Mascot (Ver 2.2, Matrix Science).

Molecular Dynamics Analysis

Molecular dynamics (MD) simulations of MEK1 were carried out with version 2.7 of NAMD (http://www.ks.uiuc.edu/Research/namd) (Phillips et al., 2005) with the CHARMM force field in release c35b5, c36a2 (http://mackerell.umaryland.edu/CHARMM_ff_params.html) (MacKerell et al., 1998). Starting geometries for the simulations were prepared from the 2.1Å crystal structure of MEK1 (containing residues 39-276 and 307-381) in the presence of ATP-γS and Mg^{2+} (PDB code: 3EQD) (Fischmann et al., 2009). All waters as well as the crystallographic sodium and calcium ions were removed. The ATP-γS was converted into ATP by manual editing of the pdb file. For the S200 and S218/222 simulations, serines were phosphorylated using SP2 patch in the NAMD utility psfgen. For all three simulations, psfgen was used to ionize histidines and add protons to the structures. The starting structures were then conjugate-gradient minimized in vacuum, after which they were solvated. Each structure was placed inside a water box extending beyond the protein atoms by 10 Å using the solvate plugin in VMD (http://www.ks.uiuc.edu/Research/vmd) (Humphrey et al., 1996) and then neutralized by the addition of an appropriate ratio of sodium and chloride ions at a final concentration of 150 mM using the Add Ions extension in VMD. The energy of the solvent was minimized in NAMD under periodic boundary conditions. Each system was then energy-minimized with progressively weaker restraints prior to the production run, which progressed for 60 ns. The simulation largely followed the protocol outlined in the NAMD tutorial available online (http://www.ks.uiuc.edu/Training/Tutorials/namd/namd-tutorial-unix-html/index.html). Molecular coordinates were written to the trajectory every 100 ps. The simulations were analyzed in VMD. Molecular graphics were prepared with PyMOL (Schrödinger LLC).

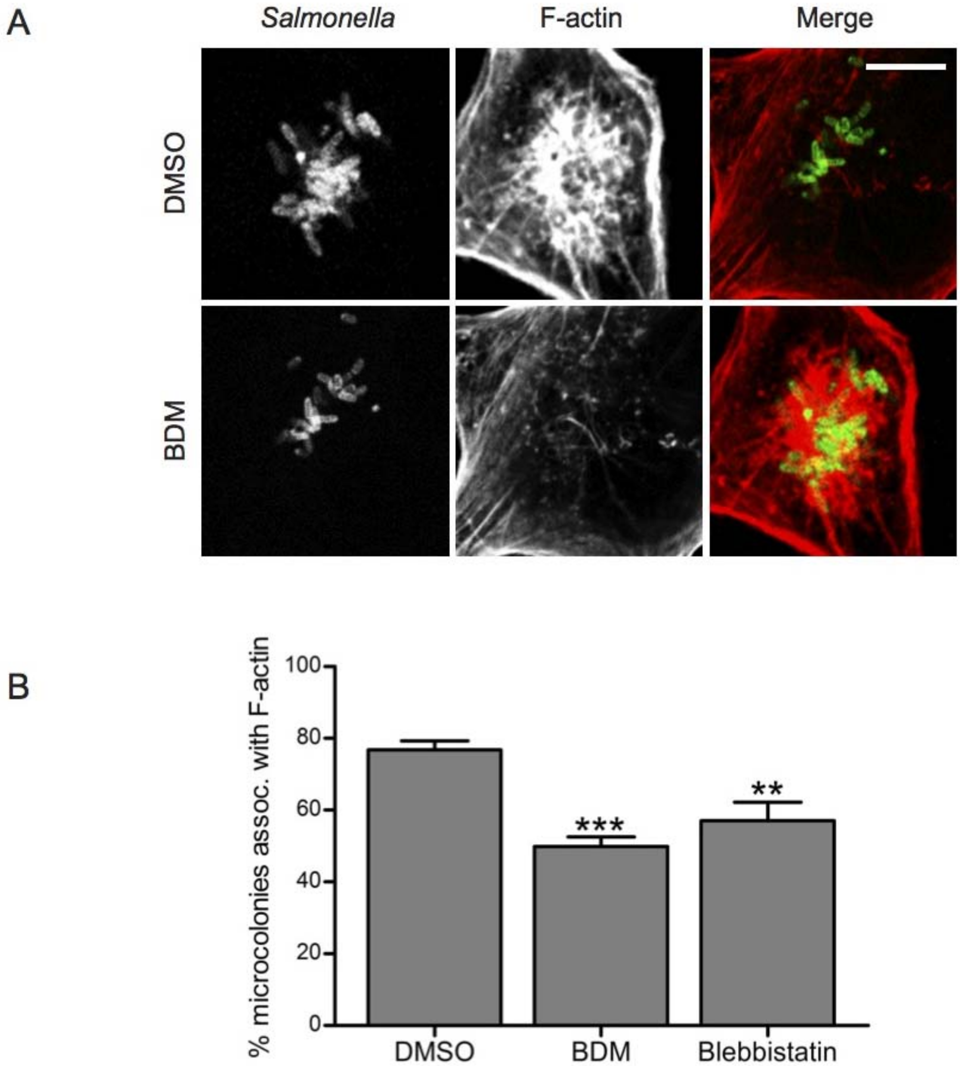

Figure S1. Blebbistatin and BDM reduce SCV association with F-actin, Related to Figure 1

Swiss 3T3 fibroblasts were infected with GFP-expressing wt *S.* Typhimurium (green) and subjected to Latrunculin B washout, then incubated in DMSO, 5 mM BDM or 25 μM Blebbistatin, for 30 min. Cells were then fixed and stained for F-actin (red).

A: Representative image of cells treated with DMSO or BDM. The scale bar represents 8 μm.

B: Percentage of *Salmonella* microcolonies associated with F-actin. Results are expressed as means ± SEM of at least four independent experiments. ⋆⋆:$p<0.01$, ⋆⋆⋆:$p<0.001$.

A

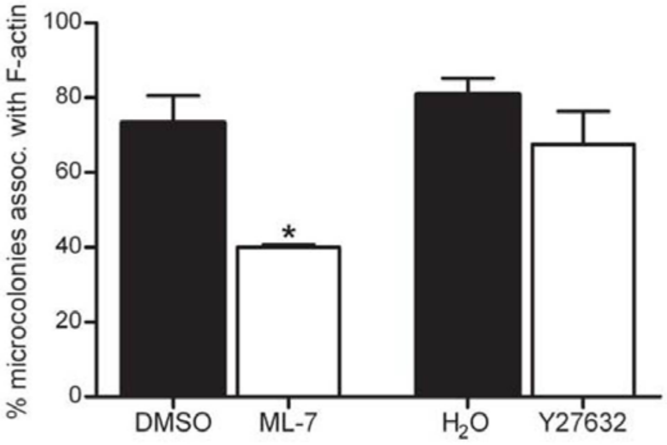
% microcolonies assoc. with F-actin
100
80
60
40
20
0
*
DMSO
ML-7
H2O
Y27632

B

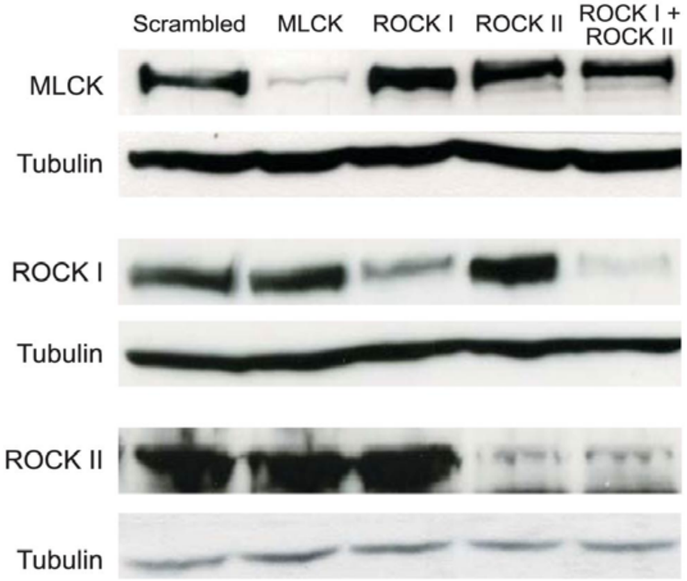
Scrambled
MLCK
ROCK I
ROCK II
ROCK I + ROCK II
MLCK
Tubulin
ROCK I
Tubulin
ROCK II
Tubulin

C

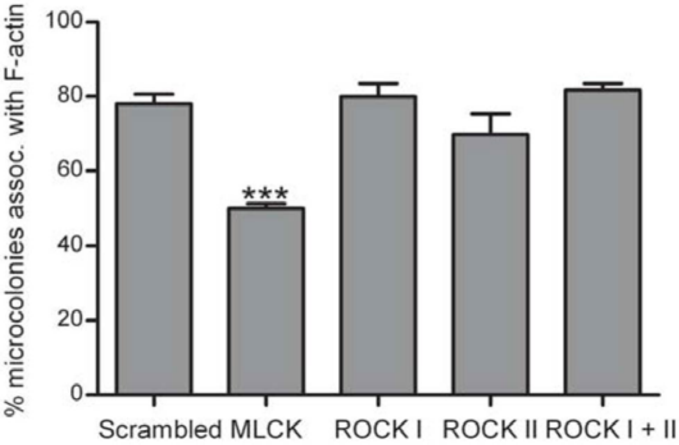
% microcolonies assoc. with F-actin
100
80
60
40
20
0
***
Scrambled
MLCK
ROCK I
ROCK II
ROCK I + II

Figure S2. ROCK isoforms are not involved in SteC-dependent F-actin reorganisation, Related to Figure 2

A: Swiss 3T3 fibroblasts were infected with wt *S.* Typhimurium and subjected to Latrunculin B washout, then incubated in DMSO, 30 μM ML-7, H_2O or 30 μM Y27632, for 30 min. The percentage of *Salmonella* microcolonies associated with F-actin was quantified. Results are expressed as means ± SEM of at least four independent experiments. ⋆:$p<0.05$.

B-C: Swiss 3T3 fibroblasts were transfected with scrambled, MLCK, ROCK I, or ROCK II siRNAs, or mixtures of these oligos, as indicated. Whole cell lysates were analysed by WB against MLCK, ROCK I or ROCK II. Membranes in (B) were probed for tubulin as a loading control. siRNA-treated cells were infected with wt *S.* Typhimurium, and bacterial microcolonies were scored for their association with F-actin. Results are expressed as means ± SEM of at least four independent experiments (C). ⋆⋆⋆:$p<0.001$.

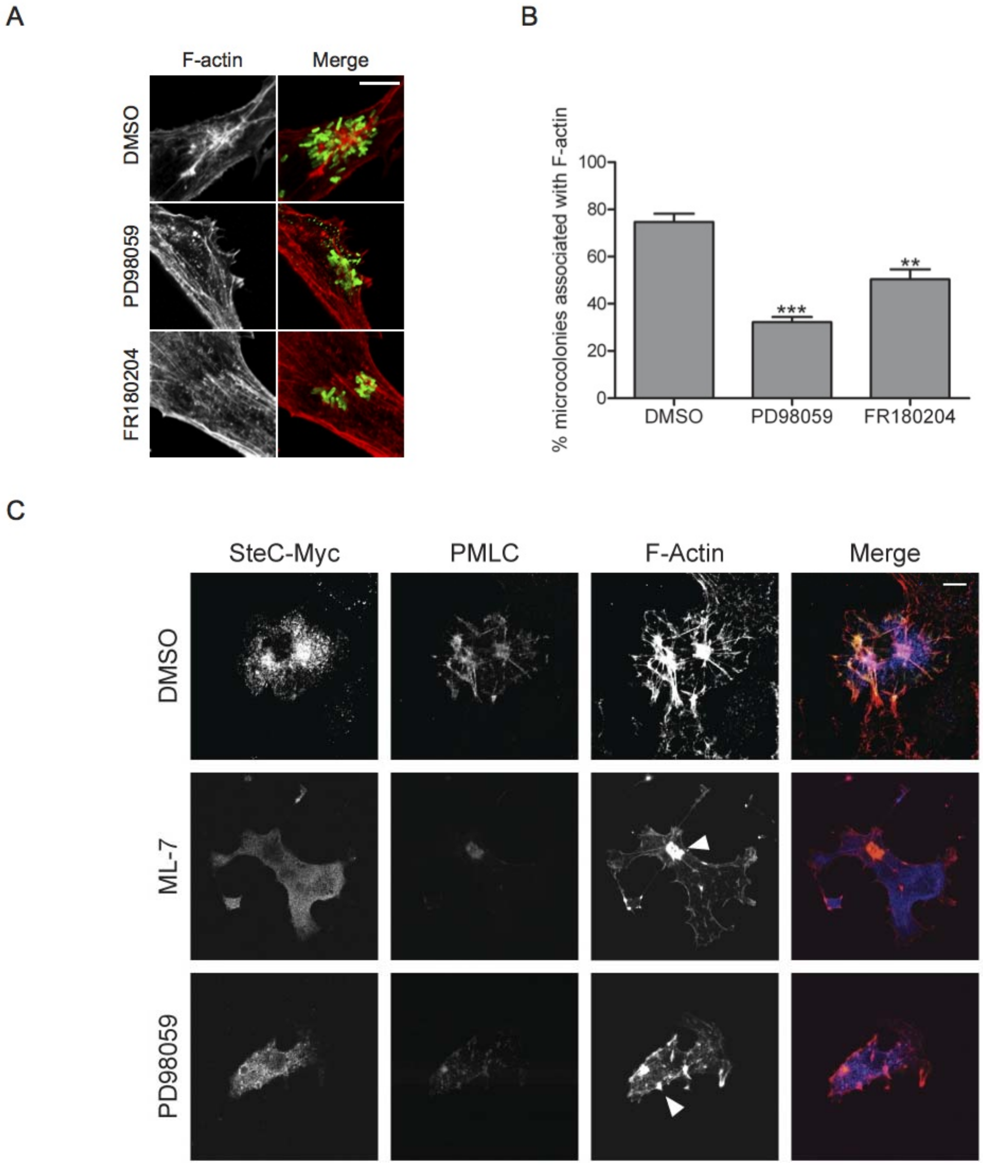

Figure S3. Effects of MEK, ERK and MLCK inhibition on SteC-dependent F-actin structures in SteC-expressing cells and *Salmonella*-infected cells, Related to Figure 3

A-B: Swiss 3T3 fibroblasts were infected with wt *S.* Typhimurium. Cells were treated with 1 µg/ml Latrunculin B, 4 h after invasion. After another 4 h, the drug was removed by thorough washing and the cells were incubated with 30 µM PD98059 (to inhibit MEK) or 20 µM FR180204 (to inhibit ERK) for 30 min. A: Representative image of cells infected with GFP-expressing wt *Salmonella* (green) and stained for F-actin (red). The scale bar represents 8 µm. B: Percentage of *Salmonella* microcolonies associated with F-actin. Results are expressed as means ± SEM of at least three independent experiments. ✩✩:p<0.01. ✩✩✩:p<0.001.

C: SteC-Myc-expressing fibroblasts were treated with MLCK (ML-7) or MEK (PD98059) inhibitors. Cells were labelled with anti-myc (blue), phospho-myosin light chain (PMLC-green) antibodies, and stained for F-actin (red). Scale bar represents 20 µm.

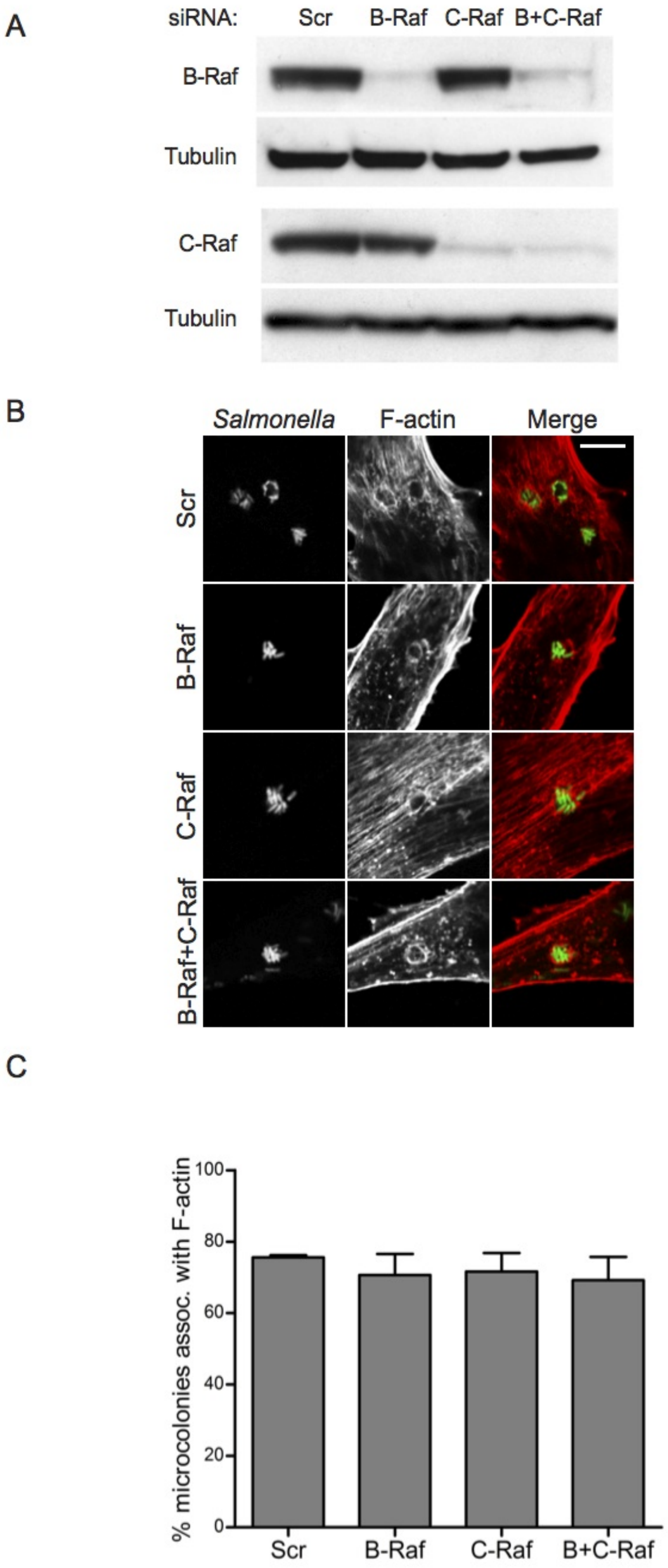
A
siRNA:
Scr
B-Raf
C-Raf
B+C-Raf
B-Raf
Tubulin
C-Raf
Tubulin
B
Salmonella
F-actin
Merge
Scr
B-Raf
C-Raf
B-Raf+C-Raf
C
% microcolonies assoc. with F-actin
100
80
60
40
20
0
Scr
B-Raf
C-Raf
B+C-Raf

Figure S4. Raf isoforms are dispensable for SteC-dependent F-actin remodelling, Related to Figure 4

A: RNAi was carried out against B-Raf, C-Raf or both proteins in Swiss 3T3 cells.
Protein levels were assessed by immunoblotting with antibodies against B-Raf, C-Raf or tubulin, which was used as a loading control.

B: Cells were subsequently infected with GFP-expressing wt *S*. Typhimurium for 8 h, fixed and stained for F-actin. The scale bar represents 8 μm.

C: The percentage of bacterial microcolonies associated with F-actin in cells transfected with the indicated siRNA oligos was quantified. Results are expressed as means ± SEM of three independent experiments.

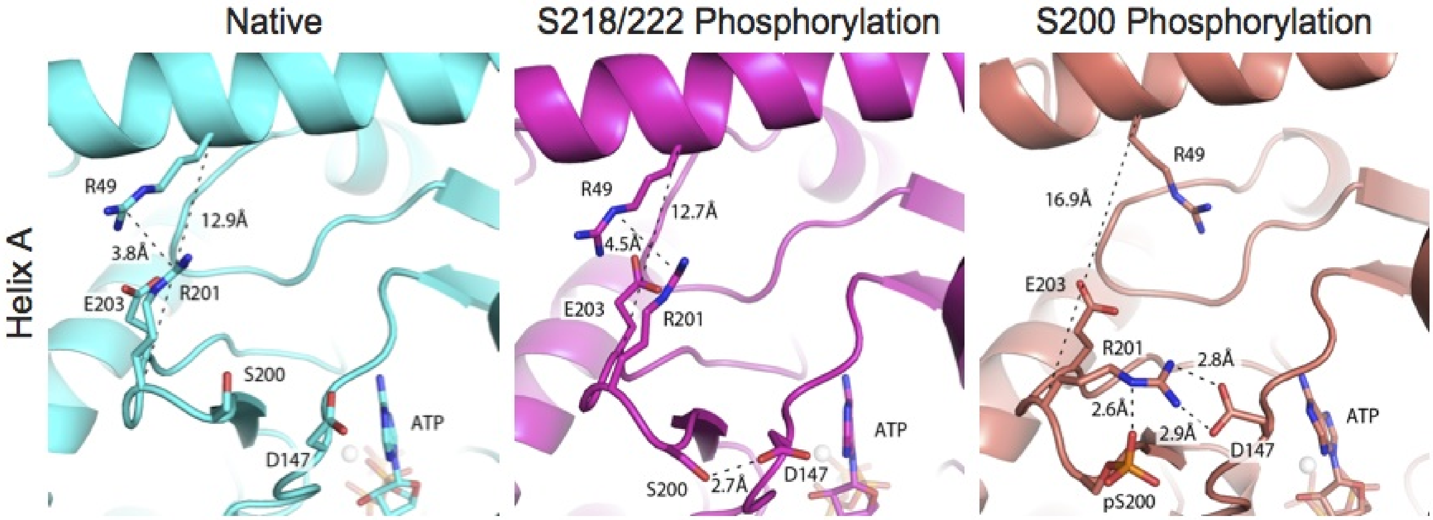

Figure S5. S200 phosphoryation induces helix A displacement, Related to Figure 5

Molecular dynamics (MD) simulations were carried out on native MEK1 (cyan), MEK1 phosphorylated on S218/222 (magenta) or MEK1 phosphorylated on S200 (salmon). All snapshots were taken at 35.6ns.

This cartoon represents the region around S200. Important residues and ATP are shown as sticks, Mg^{2+} as a white sphere. Helix A is at the top. Stabilizing interactions and the separation between the alpha carbon atoms of R49 and R201 are highlighted.

Table S1. Bacterial strains used in this study, Related to the Experimental Procedures

Name	Description	Source
12023	wt *S.* Typhimurium	National collection of type cultures (NCTC)
steC mutant	Δ*steC*::Km in 12023	(Poh et al., 2008)
ssaV mutant	Δ*ssaV*::*aphT* in 12023	(Poh et al., 2008)
Δ*steC*, p*steC2HA*	pWSK29*steC*-2HA in Δ*steC*	(Poh et al., 2008)
Δ*steC*, p*steCK256H2HA*	pWSK29*steCK256H*-2HA in Δ*steC*	(Poh et al., 2008)

Table S2. Plasmids used in this study, Related to the Experimental Procedures

Name	Description	Source
pRK5*steCmyc*	SteC tagged with N-terminal c-myc tag	(Poh et al., 2008)
pET28b*steC-6His*	SteC tagged with 6-His tag	(Poh et al., 2008)
pET28b*steCK256H-6His*	SteC tagged with 6-His tag	(Poh et al., 2008)
pCMV*HAmek1*	MEK1 with N-terminal HA tag	(Park et al., 2007)
pCMV*HAmek1S200D*	S200D MEK1 with N-terminal HA tag	This study
pCMV*HAmek1S200E*	S200E MEK1 with N-terminal HA tag	This study
pMSCV*mek1Flag*	MEK1 with C-terminal Flag tag	This study
pMSCV*mek1S200AFlag*	MEK1S200A with C-terminal Flag tag	This study

Table S3. Antibodies used in this study, Related to the Experimental Procedures

Antigen	Species	Source
Actin	Rabbit	Covance
B-Raf	Mouse	Santa Cruz
ERK1/2	Mouse	Cell Signalling
Flag	Rat	Biolegend
HA	Mouse	Covance
MEK1	Rabbit	Cell Signalling
MEK2	Rabbit	Cell Signalling
MLCK	Mouse	Sigma
Myc	Mouse	Santa Cruz
Myosin IIA heavy chain	Rabbit	Affinity Bioreagents
Myosin IIB heavy chain	Rabbit	Affinity Bioreagents
pERK1/2	Rabbit	Cell Signalling
pMEK1/2 S218/222	Rabbit	Cell Signalling
PMLC	Rabbit	Cell Signalling
C-Raf	Mouse	BD Biosciences
ROCK I	Rabbit	Chemicon International
ROCK II	Goat	Santa Cruz
Salmonella CSA-1	Goat	Kirkegaard and Perry Laboratories
β-tubulin	Mouse	Developmental Studies Hybridoma Bank

Supplemental References

Fischmann, T.O., Smith, C.K., Mayhood, T.W., Myers, J.E., Reichert, P., Mannarino, A., Carr, D., Zhu, H., Wong, J., Yang, R.S., et al. (2009). Crystal Structures of MEK1 Binary and Ternary Complexes with Nucleotides and Inhibitors. Biochemistry 48, 2661-2674.

Humphrey, W., Dalke, A., and Schulten, K. (1996). VMD: visual molecular dynamics. J Mol Graph 14, 33-38, 27-38.

MacKerell, A.D., Bashford, D., Bellott, Dunbrack, R.L., Evanseck, J.D., Field, M.J., Fischer, S., Gao, J., Guo, H., Ha, S., et al. (1998). All-Atom Empirical Potential for Molecular Modeling and Dynamics Studies of Proteins. The Journal of Physical Chemistry B 102, 3586-3616.

Park, E.R., Eblen, S.T., and Catling, A.D. (2007). MEK1 activation by PAK: a novel mechanism. Cell Signal 19, 1488-1496.

Phillips, J.C., Braun, R., Wang, W., Gumbart, J., Tajkhorshid, E., Villa, E., Chipot, C., Skeel, R.D., Kale, L., and Schulten, K. (2005). Scalable molecular dynamics with NAMD. J Comput Chem 26, 1781-1802.

Poh, J., Odendall, C., Spanos, A., Boyle, C., Liu, M., Freemont, P., and Holden, D.W. (2008). SteC is a *Salmonella* kinase required for SPI-2-dependent F-actin remodelling. Cell Microbiol 10, 20-30.

Racoosin, E.L., and Swanson, J.A. (1989). Macrophage colony-stimulating factor (rM-CSF) stimulates pinocytosis in bone marrow-derived macrophages. J Exp Med 170, 1635-1648.

Sullivan, S., Thomson, C.E., Lamont, D.J., Jones, M.A., and Christie, J.M. (2008). *In vivo* phosphorylation site mapping and functional characterization of *Arabidopsis* phototropin 1. Mol Plant 1, 178-194.

Thingholm, T.E., Jorgensen, T.J., Jensen, O.N., and Larsen, M.R. (2006). Highly selective enrichment of phosphorylated peptides using titanium dioxide. Nat Protoc 1, 1929-1935.
